# Retraction and Replacement of: ‘Consequences of partially recessive deleterious genetic variation for the evolution of inversions suppressing recombination between sex chromosomes.’

**DOI:** 10.1093/evolut/qpae066

**Published:** 2024-06-10

*Evolution*, Volume 76, Issue 6, 1 June 2022, Pages 1320–1330, https://doi.org/10.1111/evo.14496

On June 1, 2022, *Evolution* published the article entitled ‘Consequences of partially recessive deleterious genetic variation for the evolution of inversions suppressing recombination between sex chromosomes’ by Colin Olito, Suvi Ponnikas, Bengt Hansson and Jessica K. Abbott.

In March 2023, the corresponding author notified the editors of a typo in the R code version of Equation (2) that was used to run simulations. Specifically, in the code corresponding to the second line of the equation, the exponent r was inside the bracket of the left-most selection expression, so that it read $\left(1-s{\left(\ldots \right)}^{r}\right)$ instead of ${\left(1-s\left(\ldots \right)\right)}^{r}$. For unloaded inversions, r=0; therefore, the typo had the effect of zero-ing out the entire second half of the equation. Equation (2) is a denominator term, so that smaller values lead to overestimations of inversion fitness. The typo also affected initially loaded inversions, but the effect was greatest for unloaded ones. The incorrect function was used to generate Figure 1 and in the Wright-Fisher simulations presented in Figure 2, and the results led to the conclusion that small SLR-expanding inversions have elevated fixation probabilities relative to neutral expectations.

Using the corrected code, the results now show that the fixation probability of small Y-linked inversions converges to the neutral expectation of 2/N. Thus, autosomal and SLR-expanding inversions should behave similarly under many biologically plausible parameter conditions. In addition, during the reanalysis, the authors realized that the model for autosomal inversions used an equilibrium approximation assumption that was not being made in the corresponding model for SLR-expanding inversions. To ensure comparable predictions, the authors modified the model for SLR-expanding inversions to use the same equilibrium approximation assumption as for autosomal inversions, and also derived new recursions for autosomal and SLR-expanding inversions following the logic of Nei et al. (1967). In the replacement version of the manuscript, the authors have updated Figures 1 and 2 and the corresponding text in the Results section, and presented and discussed the expanded autosomal model in the main text (and in the supplementary material).

This retraction is to clarify the scholarly record and in no way suggests any misconduct on the part of the authors. The editors commend the authors for bringing the error to the journal’s attention and for their desire to present an accurate account of their study.

The new version of the manuscript underwent the peer-review process and was ultimately accepted by the handling editors at *Evolution* and has been republished at https://doi.org/10.1093/evolut/qpae060.

